# Variability of coil inductance measurements inside an interleaving structure

**DOI:** 10.1038/s41598-022-20391-5

**Published:** 2022-09-29

**Authors:** Antonio Mocholí, Ferran Mocholí, Víctor Milián-Sánchez, José Guerra-Carmenate, Miguel E. Iglesias-Martínez, Pedro Fernández de Córdoba, Juan C. Castro-Palacio, Sarira Sahu, Juan A. Monsoriu, María Lorduy, Sergio Gallardo, Gumersindo Verdú, Valeriy A. Kolombet, Victor A. Panchelyuga

**Affiliations:** 1 Traffic Control Systems Group, ITACA Institute, Universitat Politècnica de València, Camino de Vera, s/n, 46022 Valencia, Spain; 2 Institute for Industrial, Radiophysical and Environmental Safety, Universitat Politècnica de València, Camino de Vera, s/n, 46022 Valencia, Spain; 3 Grupo de Modelización Interdisciplinar, InterTech, Instituto Universitario de Matemática Pura y Aplicada, Universitat Politècnica de València, Camino de Vera, s/n, 46022 Valencia, Spain; 4 Departamento de Telecomunicaciones y Electrónica, Facultad de Ciencias Técnicas, Universidad de Pinar del Río, C/ Martí, 270, 20100 Pinar del Río, Cuba; 5 Grupo de Ingeniería Física, Escuela de Ingeniería Aeronáutica y del Espacio, Universidad de Vigo, Edif. Manuel Martínez Risco, Campus de As Lagoas, 32004 Ourense, Spain; 6 Centro de Tecnologías Físicas: Acústica, Materiales y Astrofísica, Universitat Politècnica de València, Camino de Vera, s/n, 46022 Valencia, Spain; 7 Instituto de Ciencias Nucleares, Universidad Nacional Autónoma de México, Circuito Exterior, C.U., A. Postal 70-543, 04510 Mexico, DF Mexico; 8 Departamento de Estadística e Investigación Operativa Aplicadas y Calidad, Universitat Politècnica de València, Camino de Vera, s/n, 46022 Valencia, Spain; 9 Chemical and Nuclear Engineering Department, Universitat Politècnica de València, Camino de Vera, s/n, 46022 Valencia, Spain; 10 Institute of Theoretical and Experimental Biophysics, Russian Academy of Science, Moscow Region, Pushchino, 142290 Russia

**Keywords:** Nuclear physics, Techniques and instrumentation

## Abstract

The present article is a continuation of our previous works on the anomalies in the decay rates and the capacitance measurements inside a modified Faraday cage. Here we present the anomalous variations in the measurements of inductance when a coil is placed inside an interleaving structure of metal and organic material. The fluctuation in the inductance values was found to be in the range − 0.007 to 0.018 mH. Additionally, it was observed that the temperature coefficient which was 0.0062 mH/°C initially jumped to two distinct levels, 0.0095 mH/°C and 0.0145 mH/°C, respectively. A multiple factor analysis of our results revealed a very strong correlation (R^2^ = 95.2%) between the inductance and the combination of the temperature and the relative humidity both measured inside the cage, next to the inductor.

## Introduction

It is known that many physical and chemical experiments are frequently protected from external electromagnetic disturbances by means of the well-known Faraday cages^1^. A variant of these cages is an interleaving structure, built with alternating layers of metal and organic material^2^, but unlike the conventional Faraday cages this kind of structures seem to cause modification of measurements taken inside it. So, in one of the many experiments carried out with such device, strong variations in the measurements of decay rate of Ra-226 were claimed to be observed^2^. Such a strange result was at variance with the standard exponential decay. To our knowledge, no scientific literature about the repetition of such experiment was found. In this respect, we decided to perform several experiments on decay rates measurements of several nuclides (Ra-226, Tl-204 and Sr90/I90) using a Geiger–Müller (GM) counter along with a similar enclosure. Besides, we also obtained the spectrum of Cs-137 by means of a multichannel analyser. The question on whether the GM counter tube was stable enough or not was addressed in several ways^3^. As discussed in Ref.^4^, the GM counter we used is stable and robust enough for such task. Besides, the GM counter tube environmental test provided by the manufacturer was considered (see the Appendix in Supplementary Material). Anomalous results when measuring decay rates of radionuclides were observed^3^. For example, the Ra-226 decay rates measurements increased up to 1.2%, and up to 0.8% for other nuclides. Regarding the Cs-137 spectrum measurement, the result was a displacement of the spectrum to lower energies and an increment of the photopeak of about 4%.

One hypothesis about the reason for such a variability was that the structure caused perturbations in the electronics of the detectors. Taking into account that one parameter that determines the duration of the pulses generated by the GM counter and the MCA is the time constant of the measuring circuit^5^, another experiment was carried out by using a RC low-pass filter. The measurements of the time constant yielded an increase of about 5%.

The next step was the capacitance measurement of an ultra-stable capacitor in the inside and outside of our interleaving structure. A change in the capacitance of 0.8–1% was observed. The problem became even more puzzling when Scholkmann found that our data could be correlated with cosmic weather indices like the geomagnetic activity (GMA) and with the cosmic ray induced neutron counts (CRN)^6^. To address this problem, we first looked for the circumstances under which these anomalous behaviours took place^7,8^. Subsequently in a work by Pommè and Pelzcar^9^ it was concluded that correlation with ambient humidity should also be considered. This was an indication for the improvement of our work and consequently, in a subsequent experiment^10^ we used outdoor values of the relative humidity provided by the Spanish State Agency AEMET^11^. A correlation between capacitance Cp and GMA (R^2^ = 36%) was found.

The present work is a continuation of the previous work but, with the following variations: first, measurements of inductance are carried out for a coil placed inside and outside the interleaving structure (as the coil is a very common electronic components). Second, we looked for correlations, not only with the GMA and the CRN, but also with the environmental parameters like the temperature and the relative humidity; however, this time these variables were measured inside the cage beside the device under test (DUT). The correlated variables were the inductance (L, dependent variable), and two other variables namely, the indoors ambient temperature (T_ii_) and the indoors relative humidity (RH_ii_%). This time there was no correlation, neither with the Dcx nor with the CRN, but this happened coincidentally with the *low* values of the GMA and the CRN, as described in^7^.

The outline of this work is as follows. In the next section, the “Materials and methods” used in this work are explained. Subsequently, a section with the “Results and discussion” is presented. Finally, some “Conclusions” are drawn.

## Materials and methods

**Figure. 1:**
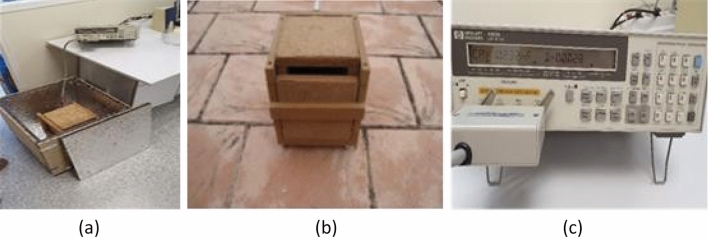
Inductance measurements inside the interleaving structure: (**a**) external cage contains the intermediate one; (**b**) 0.25 × 0.25 × 0.25 cm^3^ cage which contains an even smaller cage, (**c**) LCR measuring instrument.

The shielding surrounding the DUT was formed by an external modified Faraday cage (MFC), but this time two additional shielding structures were used one inside another (as shown Fig. 1a). The dimensions of the outer cage are 50 × 50 × 50 cm^3^. Each side of this cage contains five sheets of rough alumina and five sheets of self-adhesive elastomeric material^12^.

The intermediate cage dimensions are 25 × 25 × 25 cm^3^ with layers of galvanized iron of 1 mm thickness and 2 mm wide cork sheeting. Finally, the innermost layer is a carton box of 9 × 9 × 13 cm^3^, in which we placed the inductance. This small box was uniformly covered by a metallic mesh for electromagnetic isolation.

As before, the inductance was measured by the same instrument i.e., a 4263B HP LCR meter^13^ and the coil was a 10 mH ± 10% niquel-zinc ferrite core inductor (SBCP-80HY103HB)^14^.

The excitation signal of the LCR was set to 1 kHz and 1 V as before. Under these measurement conditions, the corresponding accuracy of the equipment was 0.08%^13^. However, we performed an analysis of variance (ANOVA) with the data to determine which results were statistically different. Figure 1 shows several images of the setup, i.e., two of the cages used and the measuring instrument.

Regarding the testing of the isolation effectiveness of the structure, a test with a mobile phone yielded that the power of the signal was reduced by 40% in the biggest box. This reduction was equivalent to 48 dBm, which is an acceptable result. As stated by some authors, to consider a Faraday cage as suitable and adequate, this value should ideally be close to 50 dBm^15^.

Regarding the correlations with the space weather and considering the study of Pommé and Pelzcar^9^, a temperature meter and a humidity meter were placed next to the inductance. The equipment chosen was the DHT22 device^16^, which was compatible with Arduino Uno Microcontroller and had a sensitivity of 0.1 °C.

### Data acquisition

Since only one LCR meter was available (Fig. 1c), inductance (L, in mH) and temperature (T, in °C) values were taken outside and inside the box at different times. First, the data were taken inside the box from Oct 27 to Nov 3, 2020. Later, data were taken outside the box from Nov 3 to Nov 11, 2020. Inductance and temperature were measured every second and every minute respectively. In this way, we could build the time series of the means corresponding to 1 min and to 1 h.

Geomagnetic activity (GMA) and cosmic ray activity (CRA) data were obtained from the same sources as mentioned in previous work^7^. In previous papers we used for GMA the classic storm index Dcx; its equivalent is the Dst index, but here we replaced it by the SYM-H geomagnetic index as it has a 1-min time resolution compared to the 1-h time resolution of Dst^17,18^. The SYM-H index is essentially the same as the Dst index with a different time resolution.

## Results and discussion

**Figure. 2:**
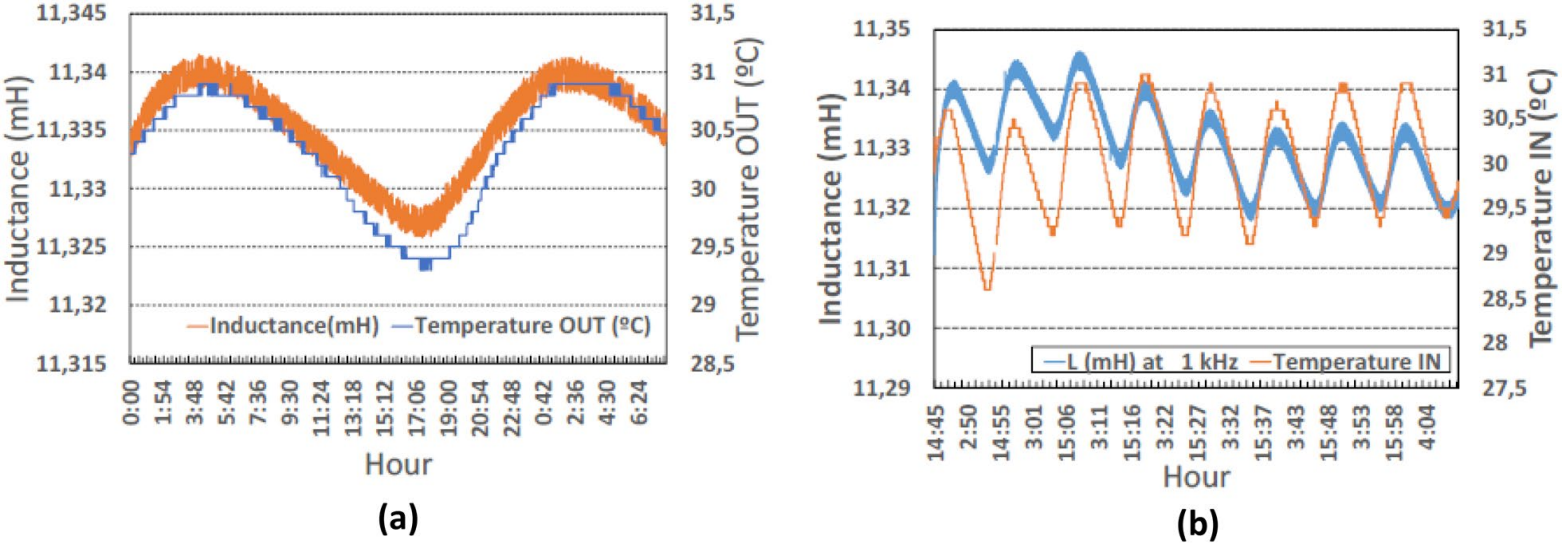
Inductance measurements vs temperature. (**a**) Plot of the inductance outside the box (from 2020 November 3 to 11) along with temperature beside the coil. The inductance follows the temperature variations. Time span of about two days for better visualize the synchronism of inductance and temperature (**b**) plot of the inductance inside the box (from 27 Oct to Nov 3); as expected, inductance deviates from temperature variations.

We measured the inductance when the system was placed indoor, but the DUT was outside the cage as shown in Fig. 2a. As expected, the inductance follows the same trend as the temperature variation (R^2^ = 94%).

However, the inductance measurements did not follow the same variation with the temperature inside the box when compared with the measurements taken outside the box. (Fig. 2b)

Table 1 shows the values of the relative maxima and minima from the graphs of both the inductance and the temperature outside the cage (measurements are taken next to the inductor) (Fig. 2a). The following quantities are included in Table 1.

- TM_j,i_ (LM_j,i_), the maximum (M) temperature (T) (inductance (L)) value of peak j, (j = 1, 2,…, 9), inside (i) *the box*. It is given in Celsius degrees (millihenries).
- Tm_j,i_ **(**L**m**_**j,i**_**)**: the minimum (m) temperature (inductance L) value of valley j inside (i) the box.

**Table 1:** Values of relative maxima and minima of temperature (T) and inductance (L) of the curves in Fig. 2a. All the peaks with the same temperature show the same inductance values. Results for the valleys are slightly different.

Outside								
**TM**_**1,o**_	**TM**_**2,o**_	**TM**_**3,o**_	**TM**_**4,o**_	**TM**_**5,o**_	**TM**_**6,o**_	**TM**_**7,o**_	**TM**_**8,o**_	**TM**_**9,o**_
30.9	30.9	30.9	30.9	30.9	30.9	30.9	30.9	30.9
**LM**_**1,o**_	**LM**_**2,o**_	**LM**_**3,o**_	**LM**_**4,o**_	**LM**_**5,o**_	**LM**_**6,o**_	**LM**_**7,o**_	**LM**_**8,o**_	**LM**_**9,o**_
11.342	11.342	11.342	11.342	11.342	11.342	11.342	11.342	11.342
**Tm**_**1,o**_	**Tm**_**2,o**_	**Tm**_**3,o**_	**Tm**_**4,o**_	**Tm**_**5,o**_	**Tm**_**6,o**_	**Tm**_**7,o**_	**Tm**_**8,o**_	**Tm**_**9,o**_
29.3	29.4	29.3	29.4	29.4	29.3	29.3	29.4	29.4
**Lm**_**1,o**_	**Lm**_**2,o**_	**Lm**_**3,o**_	**Lm**_**4,o**_	**Lm**_**5,o**_	**Lm**_**6,o**_	**Lm**_**7,o**_	**Lm**_**8,o**_	**Lm**_**9,o**_
11.326	11.326	11.326	11.326	11.326	11.326	11.325	11.326	11.326

In Table 1, the same inductance values (LM_j,o_ = 11.342 mH) correspond to the same temperature values (TM_j,o_ = 30.9 °C). Similar observation applies for valley values (third and fourth row in Table 1). However, when the coil was placed inside the cage, we observed some unexpected results, which are presented in Table 2, where the values of relative maxima of temperature (T) and inductance (L) correspond to the graphs in Fig. 2b. Specifically, the following peaks have the same value: TM_3,i_ = TM_5,i_ = TM_7,i_ = TM_8,i_ = 30.9 °C, whereas the corresponding inductance values are no longer equal to LM_j,o_ = 11.342 mH (which was the value outside), and are also different from each other (2nd row in Table 2). The differences with LM_j,o_ = 11.342 mH are shown in the third row. Moreover, the inductance LM_j,o_ = 11.342 mH (which remained constant all the time when the coil was placed outside the box), fluctuated between 11.334 and 11.346 mH when placed inside. Similar fluctuations can be observed in the inductance corresponding to the temperatures Tm_2,i_ = Tm_4,i_ = 29.2 °C and to Tm_3,i_ = Tm_6,i_ = Tm_7,i_ = 29.3 °C.

**Table 2:** Values of relative maxima and minima of the curves in Fig. 2b.

Inside							
**TM**_**1,i**_	**TM**_**2,i**_	**TM**_**3,i**_	**TM**_**4,i**_	**TM**_**5,i**_	**TM**_**6,i**_	**TM**_**7,i**_	**TM**_**8,i**_
30.6	30.5	30.9	31.0	30.9	30.7	30.9	30.9
**LM**_**1,i**_	**LM**_**2,i**_	**LM**_**3,i**_	**LM**_**4,i**_	**LM**_**5,i**_	**LM**_**6,i**_	**LM**_**7,i**_	**LM**_**8,i**_
11.342	11.345	11.346	11.341	11.337	11.334	11.335	11.334
		+ 0.004		− 0.005		− 0.007	− 0.008
Tm_1,i_	Tm_2,i_	Tm_3,i_	Tm_4,i_	Tm_5,i_	Tm_6,i_	Tm_7,i_	Tm_8,i_
28.6	29.2	29.3	29.2	29.1	29.3	29.3	29.4
**Lm**_**1,i**_	**Lm**_**2,i**_	**Lm**_**3,i**_	**Lm**_**4,i**_	**Lm**_**5,i**_	**Lm**_**6,i**_	**Lm**_**7,i**_	**Lm**_**8,i**_
11.326	11.331	11.327	11.322	11.318	11.319	11.320	11.318

**Figure. 3:**
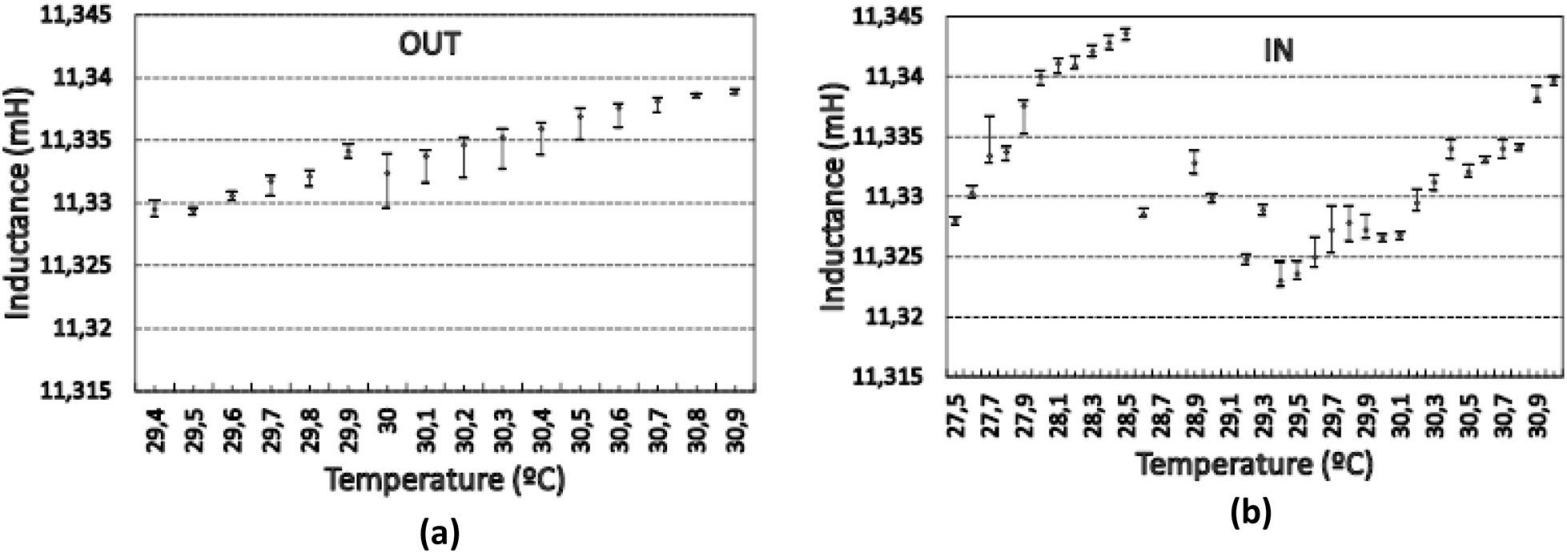
Inductance temperature coefficient. (**a**) Outside the box. (**b**) Inside the box. The increase from 27.5 to 28.5 °C took place during the days 4th and 5th of November 2020.

The observed behavior was very different in each scenario. In the first one (outside), it was clearly observed that the only variable that affected the inductance variation was the temperature. Thus, the temperature and the inductance increased and decreased at the same time. However, in the second case (inside), in addition to this phenomenon, a different behavior was apparent. This can be better understood by taking the data of Fig. 2b and rearranging them from minimum to maximum values of the temperature and performing an ANOVA analysis of the rearranged data^19^, which is shown in Fig. 3. This shows how the inductance increases with the temperature both outside and inside the MFC cage. The uncertainty bars correspond to the least significant difference (LSD), which were calculated by the statistical program Statgraphics^20^. This could be considered as an estimation of the inductance temperature coefficient (TCL).

As expected, when the coil was placed outside the cage, TCL was positive and practically linear, with a slope of about 0.0062 mH/°C (Fig. 3a). However, when the coil was placed inside the cage (Fig. 3b), the obtained data lacked the characteristic linearity of the first, which agrees with the greater variability of Fig. 3b. This time the inductance temperature coefficient shows high variation. The first ascending part of the graph (corresponding to the low temperatures) has an approximate slope of 0.0145 mH/°C. This is followed by a negative TCL of − 0.0302 mH/°C. Finally, the slope of the second ascending part is approximately 0.0095 mH/°C.

By extrapolation of the temperature values in Fig. 3a to 27.5 °C (which is the lowest temperature in Fig. 3b) a straight line can be obtained to be used as a reference. Then, the maximum difference between the inductance values in Fig. 3b and corresponding to the same temperature of the straight line in Fig. 3b can be approximately 0.025 mH (at 28.5 °C).

Besides, it is worth noting that in Fig. 3b at 28 °C the inductance is 11.34 mH and RH_ii_ ~ 35% whereas at 31 °C, L = 11.34 mH but RH_ii_ ~ 29.6%.

### Correlations

In addition to the above results, correlation between the inductance L and the other independent variables were calculated and analysed. The independent variables were:

- Temperature outdoor (T_o_); temperature indoor inside the box (T_ii_).
- Relative humidity outdoor (RH_o_); relative humidity indoor, measured inside the box (RH_ii_).
- Geomagnetic activity GMA (represented by Dcx) and,
- Cosmic ray activity (CRA) (represented by cosmic neutrons counts, N (cps)).

**Figure 4:**
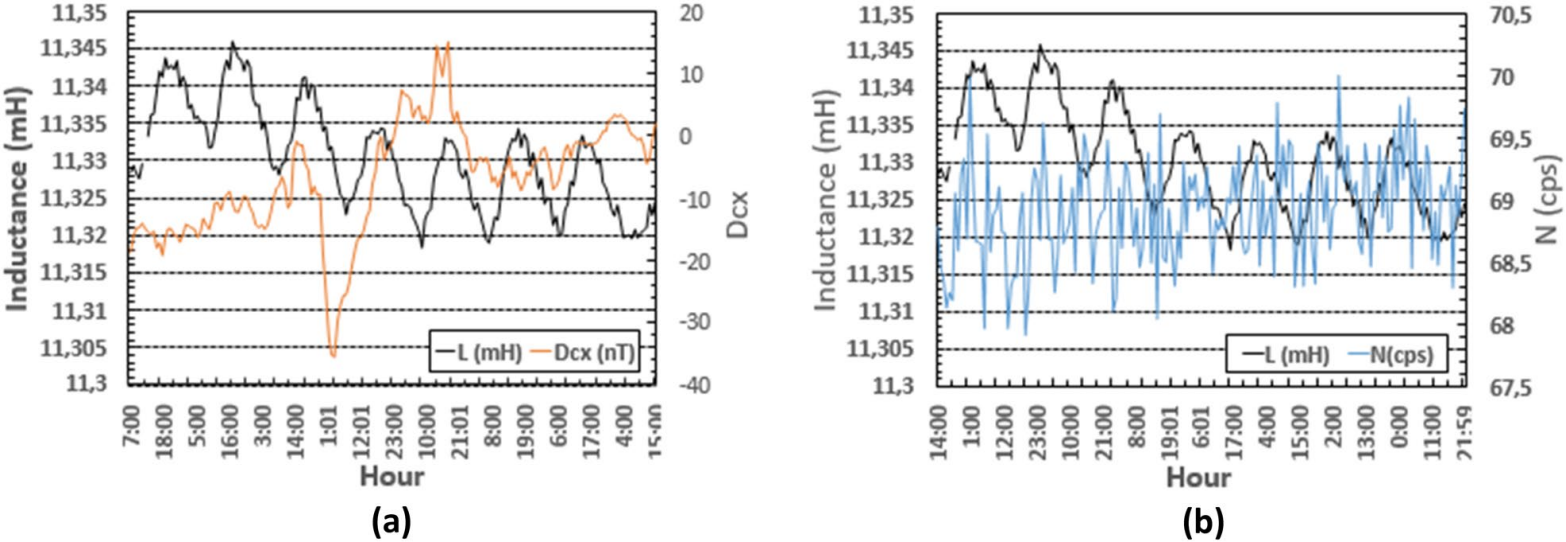
(**a**) Inductance versus Dcx, the latter ranging between − 25 and + 15 nT; (**b**) Inductance versus N, where N < 70 cps. Plots in (**a**) and (**b**) are obtained on an hourly basis (means were calculated every hour). The variability of Dst and N resembles the circumstances when there was lack of correlation with the DUT.

The values of the inductance L along with Dcx and N are presented in Fig. 4a,b on an hourly basis. This kind of variability shown by both Dcx and N was observed in^7^ to be coincident with low or no-correlation between these variables and the magnitudes under study. Specifically, Dcx remains between − 25 and + 15 nT (low level Dcx), and N remains lower than 70 cps.

On the other hand, the contribution of each variable (and/or the combination of variables) to the total variability of the inductance can be determined by simple or multiple regression analysis^21,22^ applied to the time series. The time series of L consists of the mean values taken every hour (or every minute). The first row in Table 3 shows a multiple regression coefficient of 95.4% between L and the variables considered so far when the setup is placed inside the cage, namely: T_ii_, RH_ii_, Dcx (equivalent to SYM_H) and N. A regression coefficient of 95.2% between L and T_ii_ and RH_ii_ is obtained. Finally, the correlation coefficient is small when one considers only Dcx and N.

**Table 3:** Correlation between the different variables studied.

	L vs Tii, RHii, Dcx, N	R^2^ (%)
1	L = L(Tii, RHii, Dcx, N)	95.4
2	L = L(Tii, RHii)	95.2
3	L = L(Dcx, N)	22.8

## Conclusions

The premise of this study is that the measurements of inductance of a coil can vary linearly with temperature and other external variables involved. This occurs mainly due to the increase in the resistance of the coil itself as the temperature increases. The resistance of copper wire per unit length (main material used in the construction of these electronic components) increases considerably with temperature, which causes a higher voltage to be needed to obtain the same current passing through it. Under this premise, it would have expected that the inductance value of the coil would have varied uniformly with the temperature. However, this happened only when it was measured outside the MFC. Within it, the performance was anomalous, as in the case of the capacitor and of the nuclides mentioned in^1^. The result was the observation of a variability of 0.025 mH, which is much greater than instrument accuracy (0.008 mH), and much greater than the LSD intervals calculated by the statistical program. Moreover, it was seen that the same inductance values could be measured at two different temperatures. The inductance variations were analyzed for correlations with the independent variables T_ii_, RH_ii_, Dcx and N, thus obtaining a correlation coefficient of R^2^ = 95.4%. In this specific experiment, under the current circumstances, we found a weak correlation between L and the space weather variables Dcx and N. Therefore, almost the same correlation coefficient was obtained after erasing the superfluous independent variables (Dcx, N). The low RH values would not explain a dependence of L on RH.

## Supplementary Information

Supplementary Information.

## Data Availability

The datasets used and/or analysed during the current study are available from the corresponding author or reasonably request.

## References

[1] Klinkenbusch L (2005). On the shielding effectiveness of enclosures. IEEE Trans. Electromagn. Compat..

[2] Reich W (1960). Selected Writings: An Introduction to Orgonomy.

[3] Milián-Sánchez V (2016). Anomalous effects on radiation detectors and capacitance measurements inside a modified Faraday cage. Nucl. Inst. Methods Phys. Res. A.

[4] Jenkins JH, Herminghuysen KR, Blue TE, Fischbach E, Javorsek D, Kauffman AC, Mundy DW, Sturrock PA, Talnagi JW (2012). Additional experimental evidence for a solar influence on nuclear decay rates. Astropart. Phys..

[5] Knoll GF (1999). Radiation Detection and Measurement.

[6] Scholkmann, F. *et al*. Anomalous effects on radiation detectors and capacitance measurements inside a modified Faraday cage: Correlations with space weather. *EPL* 117 (2017).

[7] Milián-Sánchez V (2020). Fluctuations in measured radioactive decay rates inside a modified Faraday cage: Correlations with space weather. Sci. Rep..

[8] Pommé S, Pelczar K (2020). On the recent claim of correlation between radioactive decay rates and space weather. Eur. Phys. J. C.

[9] Milián-Sánchez, V. *et al*. Anomalous capacitance fluctuations measurements inside a modified Faraday cage. *Eur. Phys. J. Plus***(submitted)**.

[10] Outdoor values of the relative humidity, obtained from: Agencia Estatal de Meteorología, AEMET. http://www.aemet.es

[11] Politaber autoadhesiva, Ficha técnica no. 31403-Rev. 5/20—13.01.2020. http://www.chova.com

[12] Agilent Impedance Measurement Handbook 4th Edition. https://www.slideshare.net/benlamlih/agilentimpedancemeasurements-handbook

[13] KEMET Part Number: SBCP-80HY103HB (UPIO80HY103HB0).

[14] http://codegeenprep. com/2016/06/testing-effectiveness-faraday-cage

[15] Components101.com ›sensors› dht22-pinout-specs-datasheetDHT22 Sensor Pinout, Specs, Equivalents, Circuit and Datasheet.

[16] Whenis it alright to use SYM-H as a storm index? NASA/ADS. https://ui.adsabs.harvard.edu/abs/2007AGUFMSM32A..05W/abstract

[17] Wanliss JA, Showalter KM (2006). High-resolution global storm index: Dst versus SYM-H. J. Geophys. Res. Space Phys..

[18] Tomás Miguel JM (2008). Factorial confirmatory models of Ryff's scales in a sample of elderly people. Psicothema.

[19] Statgraphics Centurion XVIII. https://www.statgraphics.com/centurion-xvii

[20] Moret-Tatay C, Gamermann D, Navarro-Pardo E, Fernández de Córdoba Castellá P (2018). ExGutils: A Python package for statistical analysis with the ex-Gaussian probability density. Front. Psychol..

[21] Moret-Tatay C, Moreno-Cid A, Argimon II, Quarti Irigaray T, Szczerbinski M, Murphy M, Vázquez-Martínez A, Vázquez-Molina J, Sáiz-Mauleón B, Navarro-Pardo E, Fernández de Córdoba P (2014). The effects of age and emotional valence on recognition memory: An ex-Gaussian components analysis. Scand. J. Psychol..

[22] Iglesias-Martínez ME, Castro-Palacio JC, Scholkmann F, Milián-Sánchez V, Fernández de Córdoba P, Mocholí-Salcedo A, Mocholí F, Kolombet VA, Panchelyuga VA, Verdú G (2020). Correlations between background radiation inside a multilayer interleaving structure, geomagnetic activity, and cosmic radiation: A fourth order cumulant-based correlation analysis. Mathematics.

