# Supplementary material for: Variability of coil inductance measurements inside an interleaving structure
Source: Sci Rep. 2022 Sep 29;12:16272. doi: 10.1038/s41598-022-20391-5 (PMC9523029; doi:10.1038/s41598-022-20391-5)
Supplement: Supplementary file 1 — Supplementary Information. [file 41598_2022_20391_MOESM1_ESM.docx]

Supplementary Material

**Appendix**

The table below is the environmental test of the used GM counter tube ZP1430 provided by the manufacturer [23]. The run time for each run was *100 seconds and operating voltage of 575 volts:*

|  | ***Temperature*** | ***Mean Recorded count*** | ***Counts increase between runs (%)*** |
| --- | --- | --- | --- |
| *Run 1.* | *23 ºC* | *170188* |  |
| *Run 2.* | *50 ºC* | *172522* | *1.37* |
| *Run 3.* | *60 ºC* | *173346* | *0.47* |
| *Run 4.* | *70 ºC* | *174405* | *0.61* |
|  |  |  |  |
| *Statistical measuring error* |  |  | *+/-0.25% for 2*sigma* |

*An increase in counts of 1,2% would correspond to a temperature increase of about 24 ºC, not to the instability of the GM counter tube.*

[23] GM counter tube ZP1430 obtained from: <http://www.centronic.co.uk/>
